# Delirium in the United States: Results From the 2023 Cross-Sectional World Delirium Awareness Day Prevalence Study

**DOI:** 10.1016/j.jaclp.2024.06.005

**Published:** 2024-06-27

**Authors:** Heidi Lindroth, Tru Byrnes, Mikita Fuchita, Breanna Hetland, Keibun Liu, Kerri Maya, Natalie S. McAndrew, Malissa A. Mulkey, Peter Nydahl, Jessica Palakshappa, Rebecca von Haken, Kevin J. Psoter, Esther S. Oh, the U.S. WDAD Study Team

**Affiliations:** Division of Nursing Research, Department of Nursing, Mayo Clinic, Rochester, MN; Center for Aging Research, Regenstrief Institute, School of Medicine, Indiana University, Indianapolis, IN; Center for Health Innovation and Implementation Science, School of Medicine, Indiana University, Indianapolis, IN; Department of Nursing, Atrium Health-Carolinas Medical Center, Charlotte, NC; Division of Critical Care, Department of Anesthesiology, University of Colorado Anschutz Medical Campus, Aurora, CO; College of Nursing, University of Nebraska Medical Center & Critical Care Division, Nebraska Medicine Omaha, NE; Critical Care Research Group, The Prince Charles Hospital, Brisbane, Australia; Institute for Molecular Bioscience (IMB), The University of Queensland, Brisbane, Queensland, Australia; Non-Profit Organization ICU Collaboration Network (ICON), Tokyo, Japan; Department of Continuing Professional Development, Sutter Health System, Sacramento, CA; University of Wisconsin-Milwaukee, School of Nursing, College of Health Professions & Sciences, Milwaukee, WI; Froedtert & the Medical College of Wisconsin, Froedtert Hospital, Milwaukee, WI; Department of Biobehavioral and Nursing Science, College of Nursing, University of South Carolina, Columbia, SC; Nursing Research, University Hospital Schleswig-Holstein, Kiel Germany; Institute of Nursing Science and Development, Paracelsus Medical University, Salzburg, Austria; Department of Internal Medicine, Section of Pulmonary, Critical Care, Allergy, and Immunologic Diseases, Wake Forest University School of Medicine, Winston-Salem, NC; Department of Anesthesiology, University Hospital Mannheim, Germany; Division of General Pediatrics, Department of Pediatrics, the Johns Hopkins University School of Medicine, Baltimore, MD; Division of Geriatric Medicine and Gerontology, Departments of Medicine, Psychiatry and Behavioral Sciences and Pathology, the Johns Hopkins University School of Medicine, Baltimore, MD; The Johns Hopkins University School of Nursing, Baltimore, MD

**Keywords:** delirium, prevalence, survey, clinical practice, health care system

## Abstract

**Background:**

Delirium is an acute brain dysfunction associated with an increased risk of mortality and future dementia.

**Objectives:**

To describe the prevalence of clinically documented delirium in the United States on World Delirium Awareness Day 2023.

**Methods:**

This is a sub-analysis of a prospective, cross-sectional, online, international survey. All health care settings were eligible, with the exception of operating rooms and outpatient clinics. Health care clinicians, administrators, and researchers completed the survey. The primary outcome was the prevalence of clinically documented delirium at 8:00 a.m. and 8:00 p.m. on March 15, 2023. Secondary outcomes were related to health care delivery. Descriptive statistics are reported. Differences between unit types (non-intensive care unit vs intensive care unit) were examined for all outcomes.

**Results:**

Ninety-one hospital units reported on 1318/1213 patients. The prevalence of clinically documented delirium was 16.4% (n = 216/1318) at 8:00 a.m. and 17.9% (n = 217/1213) at 8:00 p.m. (*P* = 0.316) and significantly differed between age groups, reported discipline, unit, and hospital types. Significant differences were identified between non-intensive care unit and intensive care unit settings in the use of deliriumrelated protocols, nonpharmacologic and pharmacologic management, educational processes, and barriers to evidence-based delirium care.

**Conclusions:**

To our knowledge, this is the first epidemiologic survey of clinically documented delirium across two time points in the United States. Delirium remains a significant burden and challenge for health care systems.

The high percentage of units using delirium management protocols suggests administrator and clinician awareness of evidence-based strategies for its detection and mitigation. We provide recommendations for future studies and quality improvement projects to improve clinical recognition and management of delirium.

## INTRODUCTION

Delirium is an acute physiological disruption of brain networks that manifests clinically as disorders of attention and cognition.^1,2^ These disorders fluctuate in severity, making accurate determinations of prevalence difficult within acutely hospitalized populations. Prevalence has been reported to range from 10–80% depending on the care setting and patient care population.^3–5^

Although delirium is often temporary and transient,^6^ it has negative short-term and long-term consequences. Delirium can compromise patient safety by increasing the risk of falls, aspiration events, and the use of physical and chemical restraints.^7–9^ Delirium is also associated with patient and caregiver distress, longer length of hospital stay, loss of function, decline in cognitive function, including incident dementia, and greater mortality.^5,10–13^ Altogether, delirium is estimated to cost over $164 billion annually in U.S. health care expenditures.^14^ Despite these adverse outcomes, delirium is frequently missed in clinical care, in part because there is limited use of validated delirium screening tools.^15–18^ Furthermore, there has been, and continues to be, inconsistent adoption of best practices to prevent and manage delirium.^15,17,19–21^

Extensive education and practice change initiatives addressing these limitations have been undertaken in hospital settings, ranging across unit types and patient care populations.^22–28^ If clinical practice adopts routine use of validated tools to detect and document delirium, its prevalence across clinical settings can be better estimated. This will enable the allocation of resources to those clinical settings most in need and facilitate targeted delirium prevention and treatment efforts. However, neither the impact of these initiatives to increase the detection and documentation of delirium nor the routine use of evidence-based tools and interventions to mitigate delirium are well documented.

To address these gaps in knowledge, an international group of delirium investigators, representing 44 countries, deployed an international one-day prevalence study on World Delirium Awareness Day (WDAD), March 15th, 2023. In our present study, we conducted a subanalysis of the data collected in the United States on WDAD. The primary objective was to report the prevalence of clinically documented delirium across institutions and settings. The secondary objective was to summarize: (i) the delirium-related protocols; (ii) delirium-related education and clinical processes; (iii) nonpharmacologic and pharmacologic management; and (iv) barriers to delirium care. To our knowledge, this is the largest study to report on the clinically documented prevalence of delirium in the United States.

## METHODS

### Site Characteristics

The present study is a subanalysis of a prospective, international prevalence survey conducted on WDAD on March 15th, 2023. Our present analysis for the United States comprises reports from 91 hospital units from all regions of the country. We did not collect patient identifiers or protected health information for the WDAD survey. Each participating site obtained institutional review board approval based on institutional policy.

### Site Participation

The web-based survey was distributed to study co-investigators at each participating health care system through email on March 14th, 2023. All unit types (intensive care unit [ICU], intermediate care unit, general care) were eligible for study inclusion, and units were selected for inclusion by the study co-investigators based on their access to data (access to electronic health record or via unit leaders). The STrengthening the Reporting of OBservational studies in Epidemiology (STROBE) reporting guidelines were followed.

### Survey Development

The web-based survey was iteratively developed, revised, and pretested with an international, interprofessional team of clinicians and researchers. The ease of survey completion was prioritized over the granularity of data.

The final survey instrument consisted of 14 sections related to the demographics of unit and hospital system (unit type, patient age group, hospital type, discipline working on unit, variable definitions displayed in eTable 1 of online supplement), clinical use of delirium detection tools, clinically documented prevalence of delirium, delirium-related protocols, education and processes, delirium management (nonpharmacologic and pharmacologic), and barriers to the use of delirium-related tools and interventions. The final survey is on the study website (wdad-study.center).

### Data Collection

All data were collected on WDAD, March 15th, 2023, at two timepoints 8:00 a.m. (± 4 hours) and 8:00 p.m. (± 4 hours). Only aggregate unit-level data were collected; therefore, patients assessed at 8:00 a.m. may or may not have been included in the 8:00 p.m. sample, depending on their length of stay.

### Outcomes

The primary outcome was the prevalence of clinically documented delirium. Survey respondents indicated how delirium was assessed on their units, which discipline (e.g., medical, surgical, mixed) was responsible for completing delirium assessments, the number of patients assessed for delirium, and the number of patients documented as delirium positive or negative. We calculated the prevalence by dividing the number of patients who screened positive by the total number of patients assessed for delirium.

Secondary outcomes were: (i) the reported use of delirium-related protocols; (ii) nonpharmacological and pharmacological management; (iii) delirium-related education and processes; and (iv) barriers to delirium care.

During the postsurvey follow-up meetings and subsequent discussions among the U.S. 2023 WDAD study co-investigators, two additional unexpected secondary outcomes emerged, namely, clinical projects spurred by the survey and lessons learned. These are described in the results section.

### Data Analysis

Respondent, hospital, and unit characteristics of participating units were summarized by descriptive statistics. Unit types (non-ICU vs ICU) were compared using Student’s t-tests and chi square or Fisher’s exact tests for continuous and categorical variables. The label “ICU” represents the survey category of “high acuity/intermediate care/intensive care units.”

We compared delirium prevalence between 8:00 a.m. and 8:00 p.m. for all the hospital units and separately for the four characteristics (age group, reported discipline on unit, unit type, and hospital type). We also compared delirium prevalence within each time point (8:00 a.m. and 8:00 p.m.) per previously defined characteristics using similar methods. A *P* value of <0.05 was considered statistically significant. No adjustments were made for multiple comparisons due to the exploratory intention of the initial prevalence study. Analyses were conducted using STATA Version 16.1 (StataCorp, College Station, TX, USA).

## RESULTS

### Site Characteristics

A total of 91 surveys were submitted from 9 states (response rate: 100%). These surveys reported on 91 unique units and 1318 patients at 8:00 a.m. and 1213 patients at 8:00 p.m. The unit type “high acuity, intermediate care unit, or intensive care unit (ICU)” was reported in 63.7% of the surveys; community hospitals were reported in 52.8% of the surveys. Further demographic characteristics are described in eTables 2 and 3 and the study flow in eFigure 1 (online supplement).

### Primary Outcome: Delirium Prevalence

The reported prevalence of delirium was 16.4% in 1318 patients at 8:00 a.m. and 17.9% in 1213 patients at 8:00 p.m. No statistically significant difference was reported between time points across units (*P* = 0.316). When examining differences between time points for each characteristic (age group, reported discipline on unit, unit type, and hospital type), only age group (18–75, >75, mixed) was statistically different at 8:00 a.m. vs 8:00 p.m. (mixed age group 8:00 a.m. 6.0% vs 8:00 p.m. 14.1%, *P* = 0.011). Table 1 displays the full results of the primary outcome analysis. Statistically significant differences were identified for each characteristic when examining differences within time points. eFigure 2 illustrates the difference in delirium prevalence between time points and unit type (non-ICU vs ICU).

In most of the 91 units (72.2%), delirium was assessed twice daily, with nurses being primarily responsible for completing this assessment (97.8%) (eTable 4). In non-ICU settings, the most frequently used delirium screening tools were the Confusion Assessment Method^29^ and the Nursing Delirium Screening Scale^30^ (18.7% and 9.9%, respectively), contrasting with the ICU category, where the most frequently used delirium screening tool was the Confusion Assessment Method-ICU^31,32^ (52.8%) (eTables 3 and 4 online supplement).

### SECONDARY OUTCOMES

#### Delirium-Related Protocols

The top three reported delirium-related, written protocols across units used were pain management (95.6%), delirium management (80.2%), and mobility and exercise (75.8%), outlined in Table 2. When considered according to unit type, differences were revealed: for non-ICU units, the top three protocols were the same as in overall findings; in contrast, in the ICU category, the top reported protocols were pain management (94.8%), the spontaneous awakening trial (81%), then the spontaneous breathing trial (77.6%), and delirium management (77.6%) were tied for third. There were significant differences between non-ICU and ICU settings in their reported use of nutrition management protocols (non-ICU: 69.7% vs ICU: 48.3%, *P* = 0.048) and ICU-specific protocols such as spontaneous awakening trial, spontaneous breathing trial, sedation management, and ICU diaries.

##### Nonpharmacologic Interventions

The top three nonpharmacological interventions across units were pain management (95.6%), verbal reorientation (94.5%), and mobilization (87.9%), as shown in Table 3. The ICU settings reported greater use than non-ICU settings for the use of physical restraints (41.4% vs 12.1%, *P* = 0.004) and bed rails (15.5% vs 0%, *P* = 0.024). In contrast, non-ICU settings reported greater use of sensory aids for vision, hearing, and mobility (75.8% vs. 53.5%, *P* = 0.035), and a higher proportion promoted non-disturbed sleep (63.6% vs 36.2%, *P* = 0.012), and informing patients about delirium (57.6% vs 20.7%, *P* < 0.001).

##### Pharmacologic Interventions

The top three pharmacological interventions were: reduction of deliriogenic drugs (46.2%), followed by melatonin use (39.5%), and evaluation of drugs by a specialist (38.5%, i.e., consultation by a delirium expert, which could include a consultant-liaison psychiatrist, neurologist, geriatrician, pharmacist, nursing, etc.). Reported melatonin use was significantly higher in non-ICU setting than in ICU settings (54.6% vs 31%, *P* = 0.027), respectively. The reported use of dexmedetomidine and midazolam was significantly higher in the ICU setting (non-ICU: 0.0% vs ICU: 48.3%, *P* < 0.001; non-ICU: 0.0% vs ICU: 12.1%, *P* = 0.046).

Survey respondents reported that pharmacological management across units was dependent on the specific symptoms of each patient’s delirium (64.8%), reported in handovers (58.2%), and a more individualized approach that depended on the patient and side effect profile (55.0%). Non-ICU and ICU settings were significantly different in their employment of a more general approach to pharmacological management category (non-ICU: 18.2%, vs ICU: 46.6%, *P* = 0.007). Further details are provided in Table 4.

##### Delirium-Related Education and Processes

The top three reported delirium-related education and processes across units were educational training about delirium (65.9%), delirium mentioned in handovers (52.8%), and communication of delirium screening rate on the unit (49.5%; Table 2). The non-ICU setting reported a significantly higher use of delirium flyers for staff than did ICU units (non-ICU: 60.6% vs ICU: 31%, *P* = 0.006) and a significantly higher presence of dedicated delirium experts known by staff (non-ICU: 42.4% vs ICU: 19%, *P* = 0.016).

##### Barriers to Delirium Care

The top barriers to the implementation of delirium care and/or use of evidence-based strategies across units were the shortage of personnel/staff (64.8%), lack of awareness (56.0%), and lack of time to educate and train staff (51.7%). Significant differences between non-ICU and ICU settings included the following: non-ICU settings more frequently reported lack of awareness (69.7% vs 48.3%, *P* = 0.048), missing knowledge about delirium (60.6% vs 36.2%, *P* = 0.025), and lack of appropriate scores for assessment of delirium (24.2% vs 8.6%, *P* = 0.041) compared to the ICU. In contrast, ICU settings reported more frequently than non-ICU settings that patients were difficult to assess (ICU 50% vs non-ICU 27.3%, *P* = 0.035), and other problems were more challenging (ICU 29.3% vs non-ICU 9.1%, *P* = 0.034). Table 5 describes results.

##### Lessons Learned From the WDAD U.S. Study Team

Through participation in survey completion and subsequent study team discussions (n = 4 online meetings and a shared brainstorming Google document), our study co-investigators recognized health care system deficiencies within their respective units. One central system deficiency was the impact of the COVID-19 pandemic on delirium management. The COVID-19 pandemic caused many experienced nurses to retire early or leave the workforce altogether, resulting in patient care being delivered by less experienced and more recent nursing graduates. These new-to-practice nurses may not have had much clinical experience or received prior education on delirium assessment and management (education for nurses was delayed during COVID-19 as direct patient care took precedence). Study sites that recognized this deficiency and the need for more delirium education shared their insights with the U.S. study team. One health care system collaborated with their education specialist team to revise the existing e-delirium educational module.

The 2023 WDAD prevalence study also gave opportunities for some of the participating hospitals to reexamine their existing delirium prevention and management programs and expand delirium assessments beyond the ICU. Study co-investigators shared their learning that each touchpoint of care across the continuum impacts the potential success of delirium prevention and management strategies. For example, one hospital added a validated delirium screening tool to nursing shift documentation on the electronic health record to increase the overall delirium screening compliance across the hospital system. Another example is eliciting feedback from clinical nurse specialists and clinical nurse leaders on their perspectives on delirium initiatives. This information will inform future opportunities to optimize delirium screening and management.

## DISCUSSION

To our knowledge, this epidemiological survey is the first to provide a clinical picture of the current delirium burden across 91 units in nine U.S. states within community and academic health care systems. The findings provide an essential benchmark for future implementation studies and quality improvement projects to measure progress toward providing evidence-based delirium care. Implications for research and clinical practice center on addressing the reported barriers, particularly the limited availability of resources (time and staff) and the lack of awareness in addressing delirium. Future initiatives should focus on changing health care culture and practice to facilitate the use of evidence-based strategies such as education and process-focused initiatives to prevent and mitigate delirium.

In 2021, there were approximately 34 million hospitalizations in the United States (the most recent year available).^33^ This is similar to prepandemic numbers with approximately 35.4 million hospitalizations in the United States in 2019.^34^ Using our clinically documented delirium rate of 17%, some 5.8 million of these patients experienced delirium during their hospitalization. This is a staggering number of patients placed at a higher risk for an adverse hospital complication, an extended hospital length of stay, institutionalization, and future dementia and mortality. These data provide an estimation of the heavy delirium burden encountered by patients, families, clinicians, and health care systems. We also note that it is possible that the delirium burden we report and the subsequent estimate of 5.8 million patients with delirium in 2021 are underestimates, as previous studies note that upward of 70% of delirium is missed in clinical care.^18,35^ This gap in clinical care can be attributed to a myriad of reasons including the barriers reported in this survey (lack of resources and limited knowledge on delirium assessment and management). Future studies may examine how novel technologies such as automated measurement can improve delirium detection in clinical care and how to improve educational strategies to increase knowledge retention on delirium assessment.

Best practice recommendations for delirium management include the avoidance or minimization of antipsychotics and benzodiazepines.^21,36–38^ These recommendations are supported by numerous meta-analyses that show inconclusive evidence to support the use of these medications.^39–42^ Despite these recommendations, over 80% of the units disclosed the use of antipsychotics, and a third reported benzodiazepine use. In fact, benzodiazepines are shown to increase the risk of delirium, particularly in the ICU.^21,43^ It is not known whether these medications were given to address patient or staff safety concerns or if nonpharmacologic prevention or management interventions were attempted before medication administration. It is promising that almost half of the units reported the reduction of deliriogenic drugs as a management strategy and the evaluation by a specialist, such as a consultant-liaison psychiatrist. These strategies align with existing best practice recommendations. Alignment with best practice recommendations and recent meta-analysis reporting on the effectiveness of nonpharmacological interventions for delirium management were also demonstrated.^36,38,39,44^ Over 70% of the units reported the use of pain management, verbal re-orientation, mobilization, adequate fluid (i.e., hydration), and liberal visiting times for families. Future quality improvement projects should work to align clinical care with best practice recommendations for the pharmacological management of delirium.

### Next Steps to Forward Progress

To advance evidence-based delirium care, an essential next step is the proactive removal of barriers. More than half of the hospital units identified key barriers: lack of staff, lack of awareness about delirium, lack of time to educate staff, and communication gaps between different disciplines. Several of the barriers relate back to education. These include the lack of awareness about delirium, a lack of knowledge about delirium, and missing attitude/delirium that is not important. Evidence-based strategies to improve delirium knowledge include face-to-face education and coaching, e-learning modules, and multicomponent interventions with the face-to-face approach demonstrating the highest effectiveness. Measuring the improvement of knowledge and change in behavior following the educational intervention over time is crucial to understand the sustainability of the effect and if subsequent interventions are needed.^45^ Another important consideration for future quality improvement projects is the incorporation of interprofessional education, as delirium is everybody’s business.^46^ Survey respondents did report on delirium awareness interventions in place on the units, and top interventions included at least one educational training on delirium, the presence of a delirium flyer, and known delirium experts on the unit. However, it is not possible to discern whether units reporting these interventions also reported fewer education-related barriers. Future studies may consider examining this relationship.

Future leaders of quality improvement projects may consider partnering with existing initiatives, such as the Age Friendly Healthcare System^47^ and the ICU Liberation Campaign,^28^ to improve patient care processes. Partnering with existing hospital-wide initiatives to share available resources to educate and train staff may ease the resource constraints and resulting barriers reported in this survey. Organizational, unit-level, and clinician-level change is challenging. The use of implementation and change management frameworks such as the Consolidated Framework for Implementation Research and Agile Implementation can help guide quality improvement and implementation projects.^48,49^ Several studies reporting on the success and failures of previous projects provide insight for future studies and should be reviewed in the planning process.^19,22,23,27,50,51^ Lastly, partnering with an expert in change management or embedding quality improvement champions in each hospital unit will help lead the team to a successful project completion. Several certificate and bootcamp opportunities exist for health care clinicians to learn quality improvement methodologies specific to delirium at annual delirium conferences.

As aforementioned, previous studies have shown the importance of an interprofessional team approach. Each team member brings a unique experience, training, and perspective to the project, optimizing its potential for success. These interprofessional members can include patient and family representatives, the clinician delivering the intervention at the bedside (i.e., the end-user), various disciplines (i.e., pharmacy, therapists [occupational, physical, speech], nursing, physicians, etc.), and specialists (i.e., consultation-liaison psychiatrists, geriatricians, neurologists, etc.). These perspectives help to localize (i.e., tailored) the project to that specific unit culture and design it to function within existing resource constraints. Interprofessional teams are well-positioned to proactively identify potential barriers to success and unintended consequences of change. Addressing these items prior to the practice change furthers the potential for success. Lastly, if the practice change is successful, each interprofessional team member contributes their expertise to develop a minimal standard operating procedure.^52^ This minimal standard operating procedure functions to sustain the practice change with a minimum set of criteria that is embedded within each respective discipline or role. Without unified agreement and a plan for continued success, the practice change will become obsolete.

### Insights From 2023 U.S. WDAD Study Team

In U.S. WDAD team discussions following survey completion, nurse leaders highlighted the continuing negative impact of the COVID-19 pandemic. Numerous studies have documented how the COVID-19 pandemic placed immense stress, workload, and burnout on nurses.^53–56^ Consequently, many experienced nurses retired or left patient care for different positions.^57^ The current workforce is largely composed of new-to-practice nurses, who, due to pandemic circumstances, likely did not receive prior delirium education and do not have senior nursing staff to precept them on delirium care concepts. To address these deficiencies, future education and training should tailor programming to the nurses’ specialty population and create opportunities to practice delirium assessment and management skills through simulation, as well as more opportunities for interprofessional delirium education.^58,59^ This type of tailored programming may provide deeper learning that may translate into improved delirium care. Additional potential project ideas generated by the U.S. WDAD 2023 study team are listed in eTable 6 in the online supplemental section.

### Limitations and Strengths

The 2023 WDAD survey results are from a convenience sample of hospitals and units, and we cannot discern whether the patients are representative of the larger population. As the survey was designed to facilitate ease of completion, it did not collect granular patient-level data such as age, biological sex, or race. It is likely that investigators more familiar with or interested in delirium were more suitable to participate than those whose focus is elsewhere in health care; therefore, the survey results may be influenced by selection bias and are not representative of health care institutions across the United States. Future surveys may address these limitations by proactively recruiting investigators who are not often involved with delirium-related research or societies. Additionally, clinically documented delirium assessments were used for this survey, and the accuracy of these assessments is not known. It is important to note that the reported practices across sites should not be taken as recommendations. Lastly, an exploratory approach was taken to describe potentially significant relationships between survey variables, and results should be interpreted as such.

While recognizing these limitations, the initial characterization of the worldwide prevalence of delirium was a major undertaking, and we believe the results add valuable knowledge to support future research and clinically based projects. The 2023 WDAD survey provided a framework for future surveys and a benchmarking point to measure future progress. Future surveys could be extended to more hospital systems and collect individual-level data to further understand individual-level factors associated with delirium.

### Future Directions

Our study is the first step toward determining the knowledge and practice gaps in the United States and implementing best practices in delirium care. We noted that many sites were eager to discuss opportunities to improve delirium management strategies. As such, we recommend the development of a tailorable delirium prevention program that provides resources and strategies to: (1) provide ongoing data monitoring on this critical health issue; (2) create sustainable prevention and management strategies; and (3) engage an interprofessional team (patients, families, and health care clinicians) in this effort to prevent and monitor for delirium.

## CONCLUSION

Delirium remains a significant challenge for inpatient care. The high percentage of nonpharmacologic protocols, like delirium management, suggests awareness of guidelines and best practices. Efforts to minimize deliriogenic drugs, optimize all multicomponent non-pharmacological interventions, and family engagement in delirium prevention efforts are underused strategies. The endorsement and adoption of evidence-based delirium assessment and management at the local unit level may promote and enhance delirium care programs. To facilitate the adoption of evidence-based delirium care, we recommended continued prevalence surveys and developing strategies to implement delirium resources.

## Supplementary Material

Online Supplement

## Figures and Tables

**TABLE 1.** Primary Outcome

	Morning 8:00 a.m.			Evening 8:00 p.m.			
	
	Total	Delirious N (%)	Nondelirious N (%)	Total	Delirious N (%)	Nondelirious N (%)	*P* value*

Overall	1318	216 (16.4)	1102 (83.6)	1213	217 (17.9)	996 (82.1)	0.316
Age groups							
<17 years	0	0	0	0	0	0	N/A
18–75 years	1090	199 (18.3)	891 (81.7)	1045	192 (18.4)	853 (81.6)	0.945
>75 years	28	5 (17.9)	23 (82.1)	26	5 (19.2)	21 (80.8)	0.897
Mixed	200	12 (6.0)	188 (94.0)	142	20 (14.1)	122 (85.9)	0.011
	*P* value within timepoint: < 0.001			*P* value within timepoint: 0.450			
Discipline							
Medical/nonsurgical	518	78 (15.1)	440 (84.9)	497	84 (16.9)	413 (83.1)	0.423
Surgical	289	20 (6.9)	269 (93.1)	262	19 (7.3)	243 (92.8)	0.880
Mixed/general	416	103 (24.8)	313 (75.2)	404	100 (24.8)	304 (75.3)	0.998
	*P* value within timepoint: < 0.001			*P* value within timepoint: <0.001			
Ward/unit							
Non-ICU	603	60 (10.0)	543 (90.1)	534	64 (12.0)	470 (88.0)	0.272
ICU (high acuity, intermediate care, or ICU)	679	156 (23.0)	523 (77.0)	645	151 (23.4)	494 (76.6)	0.851
	*P* value within timepoint: < 0.001			*P* value within timepoint: <0.001			
Hospital type							
University	178	17 (9.6)	161 (90.5)	161	24 (14.9)	137 (85.1)	0.131
University affiliated	479	74 (15.5)	405 (84.6)	459	81 (17.7)	378 (82.4)	0.365
Community	627	125 (19.9)	502 (80.1)	559	112 (20.0)	447 (80.0)	0.966
	*P* value within timepoint: 0.003			*P* value within timepoint: 0.290			

Prevalence of delirium and comparison between and within timepoints according to reported characteristics of units and hospitals.

The statistics examine the differences between the times of the assessment (see *) and within time points (e.g., compare delirium prevalence rates at 8:00 a.m. between different age groups).

The term “mixed” age group is defined as a unit that takes care of a large age range of patients. This could include pediatric, adult, and geriatric populations.

Data presented are n and (%). *P* values shown in bold indicate a significant difference between or within timepoints.

ICU = intensive care unit.

* Indicates a *P* value across timepoints, comparing delirium prevalence rates at 8:00 a.m. to 8:00 p.m.

**TABLE 2.** Comparison of Frequency of Written Protocols and Delirium Awareness Interventions in Overall Sample, Non-ICU, and ICU Settings, Organized by Decreasing Frequency in Overall Sample

	Overall n = 91	Non-ICU n = 33	ICU n = 58	*P* value

Written protocols				
Pain management (assess, prevent, and manage)	87 (95.6)	32 (97.0)	55 (94.8)	1.000
Delirium management (assess, prevent, and manage)	73 (80.2)	28 (84.9)	45 (77.6)	0.403
Mobility and exercise	69 (75.8)	28 (84.9)	41 (70.7)	0.129
Physical restraints	68 (74.7)	23 (69.7)	45 (77.6)	0.405
Nutrition management	51 (56.0)	23 (69.7)	28 (48.3)	0.048
Spontaneous awakening trial (SAT)	47 (51.7)	0 (0.0)	47 (81.0)	<0.001
Spontaneous breathing trial (SBT)	45 (49.5)	0 (0.0)	45 (77.6)	<0.001
Sedation management	38 (41.8)	5 (15.2)	33 (56.9)	<0.001
Family engagement and empowerment	32 (35.2)	10 (30.3)	22 (37.9)	0.464
Sleep	27 (29.7)	10 (30.3)	17 (29.3)	0.921
Dementia	10 (11.0)	5 (15.2)	5 (8.6)	0.338
ICU diaries	7 (7.7)	0 (0.0)	7 (12.1)	0.046
None of the above	0 (0)	0 (0)	0 (0)	N/A
Missing data	0 (0)			
Delirium awareness interventions				
At least one educational training about delirium in the last year	60 (65.9)	24 (72.7)	36 (62.1)	0.302
Delirium is mentioned in handovers	48 (52.8)	19 (57.6)	29 (50.0)	0.486
Communication of delirium screening rate on your unit	45 (49.5)	13 (39.4)	32 (55.2)	0.148
Delirium flyer for the staff	38 (41.8)	20 (60.6)	18 (31.0)	**0.006**
Delirium experts, known by the team and dedicated for delirium care	25 (27.5)	14 (42.4)	11 (19.0)	**0.016**
Informational posters about delirium	22 (24.2)	10 (30.3)	12 (20.7)	0.303
Pocket cards for delirium assessment/management	6 (6.6)	1 (3.0)	5 (8.6)	0.411
None	0 (0)	0 (0)	0 (0)	N/A
Missing data	0 (0)			

This table displays the reported delirium-related protocols and delirium awareness interventions in use on participating units. It is organized in descending order of frequency for the overall column.

Data presented are n and (%). *P* values shown in bold indicate a significant difference between non-ICU and ICU.

ICU = intensive care unit.

**TABLE 3.** Comparison of Frequency of Nonpharmacological Delirium Prevention and Therapy in Overall Sample and in Non-ICU and ICU Settings, Organized by Decreasing Frequency in Overall Sample

	Overall n = 91	Non-ICU n = 33	ICU n = 58	*P* value

Pain management	87 (95.6)	32 (97.0)	55 (94.8)	1.000
Verbal re-orientation	86 (94.5)	30 (90.9)	56 (96.6)	0.349
Mobilization (i.e., sitting on the edge of the bed or more, daytime)	80 (87.9)	32 (97.0)	48 (82.8)	0.052
Adequate fluids	71 (78.0)	29 (87.9)	42 (72.4)	0.116
Open or liberal visiting times for families (daytime)	70 (76.9)	23 (69.7)	47 (81.0)	0.217
Multi-professional team rounds	66 (72.5)	20 (60.6)	46 (79.3)	0.055
Avoidance bladder tubes/catheters	60 (65.9)	25 (75.8)	35 (60.3)	0.136
Cognitive stimulation (i.e., provision of newspapers, TV, music, other)	59 (64.8)	23 (69.7)	36 (62.1)	0.464
Family engagement	57 (62.6)	20 (60.6)	37 (63.8)	0.763
Provision glasses, hearing, mobility aids	56 (61.5)	25 (75.8)	31 (53.5)	**0.035**
Family information	54 (59.3)	19 (57.6)	35 (60.3)	0.796
Provision day and night rhythm	46 (50.6)	21 (63.6)	25 (43.1)	0.060
Multi-professional daily goals	45 (49.5)	20 (60.6)	46 (79.3)	0.055
Nondisturbed sleep (i.e., reduction of noise and light)	42 (46.2)	21 (63.6)	21 (36.2)	**0.012**
Sitter (besides the patient for longer time, mostly over hours)	42 (46.2)	15 (45.5)	27 (46.6)	0.920
Informing patients about delirium	31 (34.1)	19 (57.6)	12 (20.7)	**<0.001**
Ear plugs, sleep glasses	29 (31.9)	7 (21.2)	22 (37.9)	0.100
Physical restraints (i.e., on wrists or others)	28 (30.8)	4 (12.1)	24 (41.4)	**0.004**
Going outside the unit or ward (i.e., hospital hall, garden, sunlight)	12 (13.2)	5 (15.2)	7 (12.1)	0.676
Animal-assisted therapy	9 (9.9)	3 (9.1)	6 (10.3)	1.000
Bed boarders	9 (9.9)	0 (0.0)	9 (15.5)	**0.024**
Ground-leveled beds	7 (7.7)	2 (6.1)	5 (8.6)	1.000
Special trained experts	3 (3.3)	2 (6.1)	1 (1.7)	0.297
Activities in patient groups (i.e., singing, eating, doing exercises, other)	0 (0.0)	0 (0)	0 (0)	N/A
Missing data	0 (0)			

This table presents the reported nonpharmacological interventions that were used on the participating units. Bed boards are defined as bed rails.

Data presented are n and (%). *P* values shown in bold indicate a significant difference between non-ICU and ICU.

ICU = intensive care unit.

**TABLE 4.** Comparison of Pharmacological Management Between Overall Sample, Non-ICU, and ICU Setting, Organized by Decreasing Frequency in Overall Sample

	Overall n = 91	Non-ICU N = 33	ICU N = 58	*P* value

Majority (>50%) of patients with delirium receive				
Reducing the use of deliriogenic drugs	42 (46.2)	14 (42.4)	28 (48.3)	0.590
Melatonin	36 (39.6)	18 (54.6)	18 (31.0)	**0.027**
Evaluation by a specialist	35 (38.5)	12 (36.4)	23 (39.7)	0.756
Quetiapine	34 (37.4)	11 (33.3)	23 (39.7)	0.549
Haloperidol	29 (31.9)	7 (21.2)	22 (37.9)	0.100
Dexmedetomidine	28 (30.8)	0 (0.0)	28 (48.3)	**<0.001**
Lorazepam*	24 (26.4)	7 (21.2)	17 (29.3)	0.399
Risperidone	13 (14.3)	3 (9.1)	10 (17.2)	0.362
I do not know	13 (14.3)	6 (18.2)	7 (12.1)	0.423
Phenobarbital	8 (8.8)	1 (3.0)	7 (12.1)	0.250
Midazolam*	7 (7.7)	0 (0.0)	7 (12.1)	**0.046**
Diazepam*	7 (7.7)	1 (3.0)	6 (10.3)	0.415
Clonidine	6 (6.6)	1 (3.0)	5 (8.6)	0.411
Beta-blockers	3 (3.3)	0 (0.0)	3 (5.2)	0.551
Melperone	0 (0.0)	0 (0)	0 (0)	N/A
Levodopa	1 (11)	1 (3.0)	0 (0.0)	0.363
Distraneurin	0 (0.0)	0 (0)	0 (0)	N/A
Missing data	0 (0)			
Pharmacological management				
Depends on specific symptoms of each patient’s delirium	59 (64.8)	21 (63.6)	38 (65.5)	0.857
Is a more individual approach, depending on patients, and side effects	50 (55.0)	22 (66.7)	28 (48.3)	0.090
Is reported in handovers	53 (58.2)	17 (51.5)	36 (62.1)	0.326
Includes pharmacologists	47 (51.7)	15 (45.5)	32 (55.2)	0.372
Is discussed with families in most cases	38 (41.8)	17 (51.5)	21 (36.2)	0.155
Includes recommendations for withdrawal of delirium-related drugs	38 (41.8)	17 (51.5)	21 (36.2)	0.155
Is a more general approach, including a few pharmacological agents	33 (36.3)	6 (18.2)	27 (46.6)	**0.007**
Is based on a standard operation procedure/protocol	25 (27.5)	11 (33.3)	14 (24.1)	0.345
Is discussed with patients in most cases	24 (26.4)	9 (27.3)	15 (25.9)	0.883
Includes psychiatrist or delirium specific liaison team	10 (11.0)	4 (12.1)	6 (10.3)	1.000
None of the above	1 (11)	1 (3.0)	0 (0.0)	0.363
Missing data	0 (0)			

Data presented are n and (%). *P* values shown in bold indicate significant differences between non-ICU and ICU.

This table reports the most used medications and pharmacological interventions reported by the participating units.

ICU = intensive care unit.

* Indicates benzodiazepine.

**TABLE 5.** Comparison of Frequency of Barriers to Delirium Care Between Overall Sample, Non-ICU, and ICU, Organized by Decreasing Frequency in Overall Sample

	Overall n = 91	Non-ICU N = 33	ICU N = 58	*P* value

Shortage of personnel/staff	59 (64.8)	24 (72.7)	35 (60.3)	0.234
Lack of awareness	51 (56.0)	23 (69.7)	28 (48.3)	**0.048**
Lack of time to educate and train staff	47 (51.7)	17 (51.5)	30 (51.7)	0.985
Communication gaps between professions	46 (50.6)	15 (45.5)	31 (53.5)	0.463
Missing knowledge about delirium	41 (45.1)	20 (60.6)	21 (36.2)	**0.025**
Patients who are difficult for assessment	38 (41.8)	9 (27.3)	29 (50.0)	**0.035**
Other problems are more challenging	20 (22.0)	3 (9.1)	17 (29.3)	**0.034**
Not enough motivated staff	19 (20.9)	10 (30.3)	9 (15.5)	0.095
No cost/resources for promoting at the department	19 (20.9)	7 (21.2)	12 (20.7)	0.953
Missing attitude, delirium is not important	18 (19.8)	6 (18.2)	12 (20.7)	0.773
Lack of nonpharmacological interventions	13 (14.3)	5 (15.2)	8 (13.8)	0.859
No appropriate scores for assessment of delirium	13 (14.3)	8 (24.2)	5 (8.6)	**0.041**
We have no barriers; delirium is regularly assessed	2 (2.2)	1 (3.0)	1 (1.7)	0.596
Leadership does not support	2 (2.2)	2 (6.1)	0 (0.0)	0.129
Lack of pharmacological interventions	2 (2.2)	0 (0.0)	2 (3.5)	0.533
Interprofessional conflicts	3 (3.3)	0 (0.0)	3 (5.2)	0.551
Missing data	0 (0)			

This table describes the reported barriers to delirium care from the reporting units.

Data presented are n and (%). *P* values in bold indicate significant difference between non-ICU and ICU.

ICU = intensive care unit.

## Data Availability

The US WDAD data (de-identified) is available upon request to the corresponding author, Heidi Lindroth, Lindroth.heidi@mayo.edu.

## References

[1] chap Neurocognitive Disorders. DSM Library Diagnostic and Statistical Manual of Mental Disorders-5. Diagnostic and Statistical Manual of Mental Disorders-5. Washington, DC: American Psychiatric Association; 2013

[2] Oldham MA, Holloway RG: Delirium disorder: integrating delirium and acute encephalopathy. Neurology 2020; 95:173–178 32518149 10.1212/WNL.0000000000009949

[3] Inouye SK, Westendorp RG, Saczynski JS: Delirium in elderly people. Lancet 2014; 383:911–922 23992774 10.1016/S0140-6736(13)60688-1 PMC4120864

[4] Hshieh TT, Inouye SK, Oh ES: Delirium in the elderly. Clin Geriatr Med 2020; 36:183–199 32222295 10.1016/j.cger.2019.11.001

[5] Stollings JL, Kotfis K, Chanques G, Pun BT, Pandharipande PP, Ely EW: Delirium in critical illness: clinical manifestations, outcomes, and management. Intensive Care Med 2021; 47:1089–1103 34401939 10.1007/s00134-021-06503-1 PMC8366492

[6] Marcantonio ER: Delirium in hospitalized older adults. N Engl J Med 2017; 377:1456–1466 29020579 10.1056/NEJMcp1605501 PMC5706782

[7] Franks ZM, Alcock JA, Lam T, Haines KJ, Arora N, Ramanan M: Physical restraints and post-Traumatic stress disorder in Survivors of critical illness. A systematic review and meta-analysis. Ann Am Thorac Soc 2021; 18:689–697 33075240 10.1513/AnnalsATS.202006-738OC

[8] Dasgupta M, Brymer C: Poor functional recovery after delirium is associated with other geriatric syndromes and additional illnesses. Int Psychogeriatr 2015; 27:793–802 25519779 10.1017/S1041610214002658

[9] Sillner AY, Holle CL, Rudolph JL: The overlap between falls and delirium in hospitalized older adults: a systematic review. Clin Geriatr Med 2019; 35:221–236 30929884 10.1016/j.cger.2019.01.004

[10] Boehm LM, Jones AC, Selim AA, : Delirium-related distress in the ICU: a qualitative meta-synthesis of patient and family perspectives and experiences. Int J Nurs Stud 2021; 122, 104030 34343884 10.1016/j.ijnurstu.2021.104030 PMC8440491

[11] Khan BA, Perkins AJ, Gao S, : The confusion assessment method for the ICU-7 delirium severity scale: a novel delirium severity instrument for use in the ICU. Crit Care Med 2017; 45:851–857 28263192 10.1097/CCM.0000000000002368 PMC5392153

[12] Goldberg TE, Chen C, Wang Y, : Association of delirium with long-term cognitive decline: a meta-analysis. JAMA Neurol 2020; 77:1373–1381 32658246 10.1001/jamaneurol.2020.2273 PMC7358977

[13] Aung Thein MZ, Pereira JV, Nitchingham A, Caplan GA: A call to action for delirium research: meta-analysis and regression of delirium associated mortality. BMC Geriatr 2020; 20:325 32894065 10.1186/s12877-020-01723-4 PMC7487610

[14] Leslie DL, Marcantonio ER, Zhang Y, Leo-Summers L, Inouye SK: One-year health care costs associated with delirium in the elderly population. Arch Intern Med 2008; 168:27–32 18195192 10.1001/archinternmed.2007.4 PMC4559525

[15] Pisani MA, Devlin JW, Skrobik Y: Pain and delirium in critical illness: an exploration of key 2018 SCCM PADIS guideline evidence gaps. Semin Respir Crit Care Med 2019; 40:604–613 31826261 10.1055/s-0039-1698809

[16] Hoch J, Bauer JM, Bizer M, Arnold C, Benzinger P: Nurses’ competence in recognition and management of delirium in older patients: development and piloting of a self-assessment tool. BMC Geriatr 2022; 22:879 36402941 10.1186/s12877-022-03573-8 PMC9675220

[17] Oxenboll-Collet M, Egerod I, Christensen V, Jensen J, Thomsen T: Nurses’ and physicians’ perceptions of Confusion Assessment Method for the intensive care unit for delirium detection: focus group study. Nurs Crit Care 2018; 23:16–22 27596941 10.1111/nicc.12254

[18] Steis MR, Fick DM: Are nurses recognizing delirium? A systematic review. J Gerontol Nurs 2008; 34:40–48 10.3928/00989134-20080901-12 18795564

[19] Mudge AM, McRae P, Young A, : Implementing a ward-based programme to improve care for older inpatients: process evaluation of the cluster randomised CHERISH trial. BMC Health Serv Res 2023; 23:668 37344776 10.1186/s12913-023-09659-2 PMC10283300

[20] van de Steeg L, Langelaan M, Ijkema R, Nugus P, Wagner C: Improving delirium care for hospitalized older patients. A qualitative study identifying barriers to guideline adherence. J Eval Clin Pract 2014; 20:813–819 25081423 10.1111/jep.12229

[21] Devlin JW, Skrobik Y, Gélinas C, : Clinical practice guidelines for the prevention and management of pain, agitation/sedation, delirium, immobility, and sleep disruption in adult patients in the ICU. Crit Care Med 2018; 46:e825–e873 30113379 10.1097/CCM.0000000000003299

[22] Balas MC, Pun BT, Pasero C, : Common challenges to effective ABCDEF bundle implementation: the ICU liberation campaign experience. Crit Care Nurse 2019; 39:46–60 30710036 10.4037/ccn2019927

[23] Angel C, Brooks K, Fourie J: Standardizing management of adults with delirium hospitalized on medical-surgical Units. Perm J 2016; 20:16–1002 10.7812/TPP/16-002 PMC5101087 27644045

[24] Bush SH, Skinner E, Lawlor PG, : Adaptation, implementation, and mixed methods evaluation of an interprofessional modular clinical practice guideline for delirium management on an inpatient palliative care unit. BMC Palliat Care 2022; 21:128 35841014 10.1186/s12904-022-01010-6 PMC9287908

[25] Hshieh TT, Yang T, Gartaganis SL, Yue J, Inouye SK: Hospital elder life program: systematic review and meta-analysis of effectiveness. Am J Geriatr Psychiatry 2018; 26:1015–1033 30076080 10.1016/j.jagp.2018.06.007 PMC6362826

[26] Pun BT, Balas MC, Barnes-Daly MA, : Caring for critically ill patients with the ABCDEF bundle: results of the ICU liberation collaborative in over 15,000 adults. Crit Care Med 2019; 47:3–14 30339549 10.1097/CCM.0000000000003482 PMC6298815

[27] Stollings JL, Devlin JW, Pun BT, : Implementing the ABCDEF bundle: top 8 questions asked during the ICU liberation ABCDEF bundle improvement collaborative. Crit Care Nurse 2019; 39:36–45 30710035 10.4037/ccn2019981

[28] ICU liberation. https://www.sccm.org/iculiberation. [Accessed 13 July 2023]

[29] Inouye SK, van Dyck CH, Alessi CA, Balkin S, Siegal AP, Horwitz RI: Clarifying confusion: the confusion assessment method. A new method for detection of delirium. Ann Intern Med 1990; 113:941–948 2240918 10.7326/0003-4819-113-12-941

[30] Gaudreau J-D, Gagnon P, Harel F, Tremblay A, Roy M-A: Fast, systematic, and continuous delirium assessment in hospitalized patients: the nursing delirium screening scale. J Pain Symptom Manag 2005; 29(4):368–375 10.1016/j.jpainsymman.2004.07.009 15857740

[31] Ely EW, Inouye SK, Bernard GR, : Caring for the critically ill patient. Delirium in mechanically ventilated patients: validity and reliability of the Confusion Assessment Method for the Intensive Care Unit (CAM-ICU). JAMA, J Am Med Assoc 2001; 286:2703 10.1001/jama.286.21.2703 11730446

[32] Ely EW, Margolin R, Francis J, : Evaluation of delirium in critically ill patients: validation of the confusion assessment method for the intensive care Unit (CAM-ICU). Crit Care Med 2001; 29:1370–1379 11445689 10.1097/00003246-200107000-00012

[33] Association AH: 2021 AHA annual survey. American hospital association. https://www.aha.org/statistics/fast-facts-us-hospitals. [Accessed 29 December 2023]

[34] Statistical Brief #300: Changes in Hospitalizations and In-Hospital Deaths in the Initial Period of the COVID-19 Pandemic (April-December 2020), 38 States and DC. Agency for Healthcare Research and Quality (US); 2022 36622928

[35] van Velthuijsen EL, Zwakhalen SMG, Mulder WJ, Verhey FRJ, Kempen G: Detection and management of hyperactive and hypoactive delirium in older patients during hospitalization: a retrospective cohort study evaluating daily practice. Int J Geriatr Psychiatry 2018; 33:1521–1529 28194812 10.1002/gps.4690 PMC6221174

[36] Updated Delirium Clinical Care Standard: 2021 (Australian Commission on Safety and Quality in Health Care). 2021

[37] Yue J, Tabloski P, Dowal SL, Puelle MR, Nandan R, Inouye SK: NICE to HELP: operationalizing National Institute for Health and Clinical Excellence guidelines to improve clinical practice. J Am Geriatr Soc 2014; 62:754–761 24697606 10.1111/jgs.12768 PMC4020349

[38] Soiza RL, Myint PK: The Scottish Intercollegiate guidelines network (SIGN) 157: guidelines on risk reduction and management of delirium. Medicina (Kaunas) 2019; 55:491 31443314 10.3390/medicina55080491 PMC6722546

[39] Burry LD, Cheng W, Williamson DR, : Pharmacological and non-pharmacological interventions to prevent delirium in critically ill patients: a systematic review and network meta-analysis. Intensive Care Med 2021; 47:943–960 34379152 10.1007/s00134-021-06490-3 PMC8356549

[40] Li Y, Ma J, Jin Y, : Benzodiazepines for treatment of patients with delirium excluding those who are cared for in an intensive care unit. Cochrane Database Syst Rev 2020; 2, Cd012670 32108325 10.1002/14651858.CD012670.pub2 PMC7047222

[41] Shen YZ, Peng K, Zhang J, Meng XW, Ji FH: Effects of Haloperidol on delirium in adult patients: a systematic review and meta-analysis. Med Princ Pract 2018; 27:250–259 29518791 10.1159/000488243 PMC6062716

[42] Burry L, Mehta S, Perreault MM, : Antipsychotics for treatment of delirium in hospitalised non-ICU patients. Cochrane Database Syst Rev 2018; 6, Cd005594 29920656 10.1002/14651858.CD005594.pub3 PMC6513380

[43] Pun BT, Badenes R, Heras LCG, : Prevalence and risk factors for delirium in critically ill patients with COVID-19 (COVID-D): a multicentre cohort study. Lancet Respir Med 2021; 9:239–250 33428871 10.1016/S2213-2600(20)30552-X PMC7832119

[44] Burton JK, Craig L, Yong SQ, : Non-pharmacological interventions for preventing delirium in hospitalised non-ICU patients. Cochrane Database Syst Rev 2021; 11, Cd013307 34826144 10.1002/14651858.CD013307.pub3 PMC8623130

[45] Lee SY, Fisher J, Wand APF, : Developing delirium best practice: a systematic review of education interventions for healthcare professionals working in inpatient settings. Eur Geriatr Med 2020; 11:1–32 10.1007/s41999-019-00278-x 32297244

[46] iDelirium TIFoDS: Delirium Day. World delirium awareness Day. Available from: https://www.deliriumday.com/. [Accessed 7 June 2024]

[47] Improvement IfH: Age-friendly health systems overview. Institute for healthcare improvement. Available from: https://www.ihi.org/initiatives/age-friendly-health-systems. [Accessed 29 December 2023]

[48] Damschroder LJ, Reardon CM, Widerquist MAO, Lowery J: The updated consolidated framework for implementation research based on user feedback. Implement Sci 2022; 17:75 36309746 10.1186/s13012-022-01245-0 PMC9617234

[49] Boustani M editors. [S.l.]. In: Azar J, Solid CA, (eds) Agile Implementation : A Model for Implementing Evidence-Based Healthcare Solutions into Real-World Practice to Achieve Sustainable Change. Morgan James Publishing; 2020

[50] Trogrlic Z, van der Jagt M, Bakker J, : A systematic review of implementation strategies for assessment, prevention, and management of ICU delirium and their effect on clinical outcomes. Crit Care 2015; 19:157 25888230 10.1186/s13054-015-0886-9 PMC4428250

[51] Verweij L, Oesch S, Naef R: Tailored implementation of the FICUS multicomponent family support intervention in adult intensive care units: findings from a mixed methods contextual analysis. BMC Health Serv Res 2023; 23:1339 38041092 10.1186/s12913-023-10285-1 PMC10693161

[52] Boustani M, Alder CA, Solid CA: Agile implementation: a blueprint for implementing evidence-based healthcare solutions. J Am Geriatr Soc 2018; 66:1372–1376 29513360 10.1111/jgs.15283

[53] Christianson J, McCarthy M, Sommers-Olson B, Guttormson J, Johnson NL: Community or commodity? Perceived nurse support during the COVID-19 pandemic. Nurs Manage 2023; 54:44–53 10.1097/nmg.0000000000000079 38032808

[54] Guttormson JL, Calkins K, McAndrew N, Fitzgerald J, Losurdo H, Loonsfoot D: Critical care nurses’ experiences during the COVID-19 pandemic: a US National survey. Am J Crit Care 2022; 31:96–103 34704108 10.4037/ajcc2022312

[55] Dohrn J, Ferng YH, Shah R, Diehl E, Frazier L: Addressing mental and emotional health concerns experienced by nurses during the COVID-19 pandemic. Nurs Outlook 2022; 70:81–88 34503838 10.1016/j.outlook.2021.07.009 PMC8809193

[56] McAndrew NS, Rosa WE, Moore KM, : Sprinting in a Marathon: nursing staff and nurse leaders make meaning of practicing in COVID-19 devoted Units pre-Vaccine. SAGE Open Nurs 2023; 9, 23779608231165688 37008557 10.1177/23779608231165688 PMC10052614

[57] Tolksdorf KH, Tischler U, Heinrichs K: Correlates of turnover intention among nursing staff in the COVID-19 pandemic: a systematic review. BMC Nurs 2022; 21:174 35787700 10.1186/s12912-022-00949-4 PMC9252069

[58] Lee SY, Fisher J, Wand APF, : Developing delirium best practice: a systematic review of education interventions for healthcare professionals working in inpatient settings. Eur Geriat Med 2020; 11:1–32 10.1007/s41999-019-00278-x 32297244

[59] Kamdar BB, Makhija H, Cotton SA, : Development and evaluation of an intensive care unit video series to educate staff on delirium detection. ATS Sch 2022; 3:535–547 36726713 10.34197/ats-scholar.2022-0011OC PMC9885989

