# Supplementary material for: Delirium in the United States: Results From the 2023 Cross-Sectional World Delirium Awareness Day Prevalence Study
Source: J Acad Consult Liaison Psychiatry. Author manuscript; Available in PMC 2025 May 8. (PMC12061337; doi:10.1016/j.jaclp.2024.06.005)
Supplement: Online Supplement [file NIHMS2067739-supplement-Online_Supplement.docx]

**Online Supplemental Section**

**TITLE**: Delirium in the United States: Results from the cross-sectional 2023 World Delirium Awareness Day prevalence study

**AUTHORS**: Heidi Lindroth, PhD, RN^1-3^; Tru Byrnes, DNP, RN^4^; Mikita Fuchita, MD^5^; Breanna Hetland, PhD, RN, CCRN^6^; Keibun Liu, PhD, MD^7-9^; Kerri Maya, PhD(c), MSL, RN, NPD-BC^10^; Natalie S McAndrew, PhD, RN, ACNS-BC, CCRN-K^11,12^; Malissa A. Mulkey, PhD, APRN, CCNS, CCRN, CNRN^13^; Peter Nydahl PhD, RN^14,15^; Jessica Palakshappa, MD, MS^16^; Rebecca von Haken^17^; MD, Kevin J Psoter, PhD, MPA^18^; Esther S Oh, MD, PhD^19,20^ and the U.S. WDAD Study Team

**AFFILIATIONS**:

^1^ Division of Nursing Research, Department of Nursing, Mayo Clinic, Rochester, MN, USA

^2^ Center for Aging Research, Regenstrief Institute, School of Medicine, Indiana University, Indianapolis, IN, USA

^3^Center for Health Innovation and Implementation Science, School of Medicine, Indiana University, Indianapolis, IN, USA

^4^Department of Nursing, Atrium Health-Carolinas Medical Center, Charlotte, NC, USA

^5^Department of Anesthesiology, Division of Critical Care, University of Colorado Anschutz Medical Campus, Aurora, CO, USA

^6^College of Nursing, University of Nebraska Medical Center & Critical Care Division, Nebraska Medicine Omaha, NE, USA

^7^Critical Care Research Group, The Prince Charles Hospital, Brisbane, Australia

^8^Institute for Molecular Bioscience (IMB), The University of Queensland, Brisbane, Queensland, Australia

^9^Non-Profit Organization ICU Collaboration Network (ICON), Tokyo, Japan

^10^Professional Affiliation: Department of Continuing Professional Development, Sutter Health System, Sacramento, CA, USA.

^11^University of Wisconsin-Milwaukee, School of Nursing, College of Health Professions & Sciences, Milwaukee, WI USA

^12^Froedtert & the Medical College of Wisconsin, Froedtert Hospital, Milwaukee, WI, USA

^13^Department of Biobehavioral and Nursing Science, College of Nursing, University of South Carolina, Columbia, SC, USA

^14^Nursing Research, University Hospital Schleswig-Holstein, Kiel Germany

^15^Institute of Nursing Science and Development, Paracelsus Medical University, Salzburg, Austria

^16^Department of Internal Medicine, Section of Pulmonary, Critical Care, Allergy, and Immunologic Diseases, Wake Forest University School of Medicine, Winston-Salem, NC, USA

^17^Department of Anesthesiology, University Hospital Mannheim, Germany

^18^Division of General Pediatrics, Department of Pediatrics, the Johns Hopkins University School of Medicine, Baltimore, MD, USA

^19^Division of Geriatric Medicine and Gerontology, Departments of Medicine, Psychiatry and Behavioral Sciences and Pathology, the Johns Hopkins University School of Medicine, Baltimore, MD, USA

^20^The Johns Hopkins University School of Nursing, Baltimore, MD, USA

**Corresponding Author**:

Heidi Lindroth, Ph.D., R.N.

200 First Street SW

Rochester MN 55902

[Lindroth.heidi@mayo.edu](mailto:Lindroth.heidi@mayo.edu)

**FUNDING**: NIA/NIH K23AG076662 (HL); NINR/NIH R41NR020458 (BH), Gordon and Betty Moore Foundation (GBF9048) (BH); Oncology Nursing Foundation (RE03 Research Grant, NSM), the Cambia Health Foundation Sojourns Scholar Leadership Program (2021 Scholar, NSM); NIA/NIH R01AG076525, R01AG057725, R01AG05766 (ESO); NIH T32 Mentored Research Training Award (NIGMS/NIH GM135169, MF), The Agile Nudge University Mentoring Program (NIA/NIH 1R25 AG078136, MF), K23AG073529 (JP)


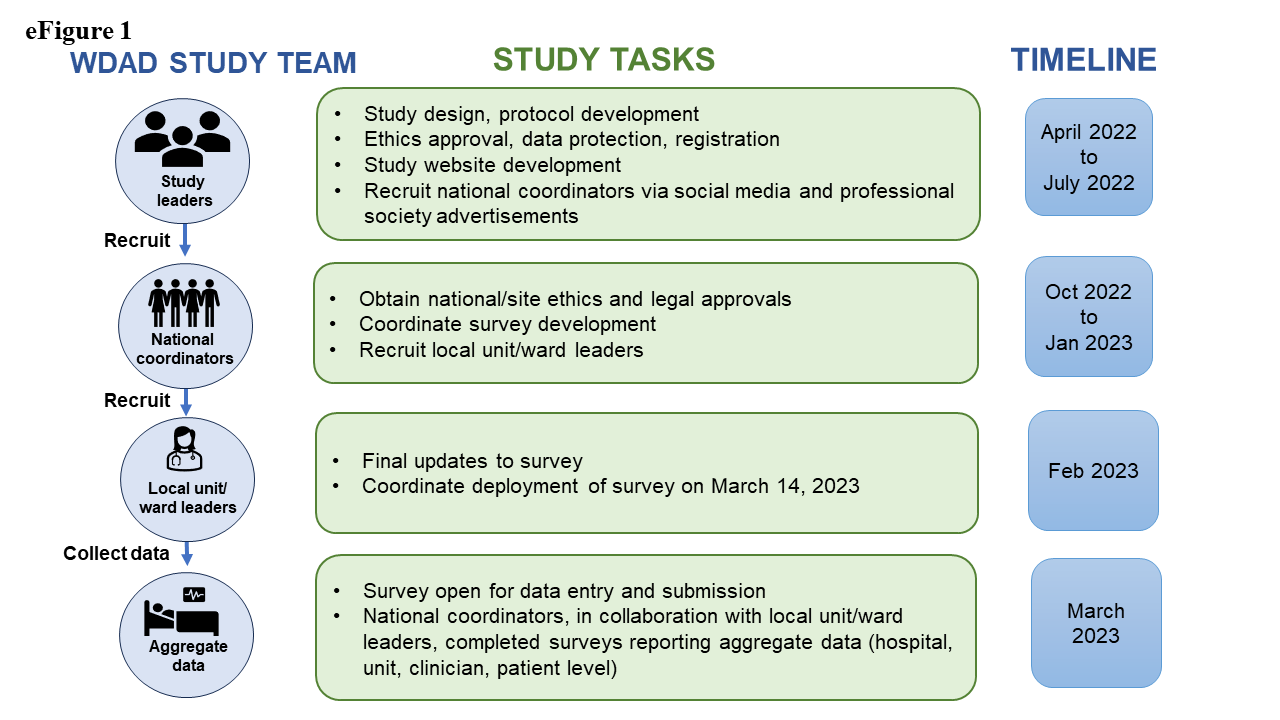


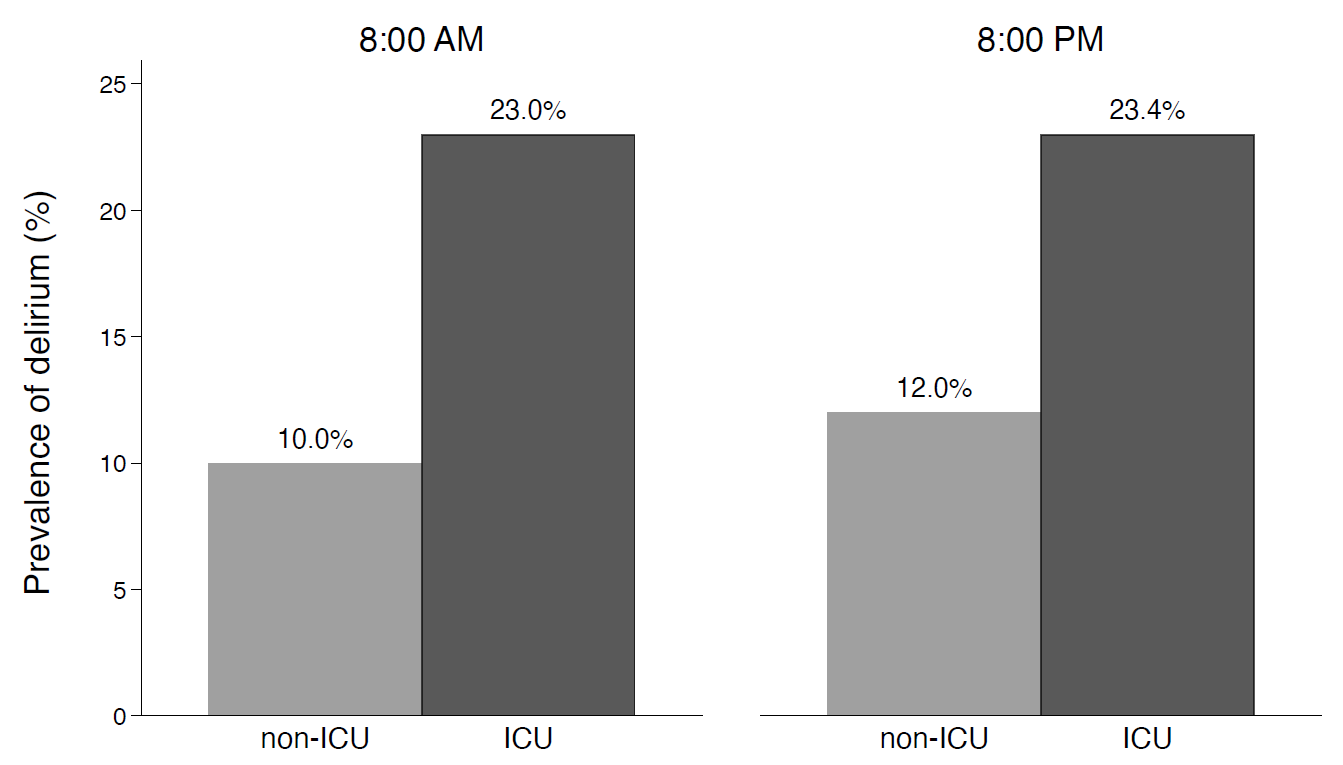


eFigure 2 illustrates the differences in delirium prevalence between non-ICU and ICU settings across the two timepoints.

**eFigure 2**

| **eTable 1. Demographic survey items and answer choices from WDAD 2023 Survey** | |
| --- | --- |
| Survey Item | Answer Options |
| Unit type | General,  High acuity/intermediate care/intensive care |
| Age group | <17  18-75  >75  Mixed |
| Hospital type | Community  Academic  Mixed |
| Discipline working on unit | Medical/Non-Surgical  Surgical  Palliative  Respiratory/weaning  Rehabilitation  Long term care  Mixed/General  Other |
| eTable 1 displays the demographic characteristics surveyed in the WDAD 2023 worldwide prevalence study. The term “mixed” age group is defined as a unit that cares for a large age range of patients. This could include pediatric, adult, and geriatric populations. The final survey is available on the study website ([wdad-study.center](http://www.wdad-study.center/)). | |

| **eTable 2. Sociodemographic data of survey respondents**.  Data presented are n and (%) from 23 participants reporting on 91 units. | |
| --- | --- |
| **Profession** | |
| Nurse | 9 (39.1) |
| Manager | 6 (26.1) |
| Researcher | 4 (17.4) |
| Physician | 3 (13.0) |
| Other | 1 (4.4) |
| Assistant | 0 (0) |
| Lecturer | 0 (0) |
| Nutritionist/dietician | 0 (0) |
| Occupational therapist | 0 (0) |
| Physical therapist | 0 (0) |
| Pharmacist | 0 (0) |
| Respiratory therapist | 0 (0) |
| Speech and swallow therapist | 0 (0) |
| Technician | 0 (0) |
| Missing data | 0 |
| **Leadership position on reported unit** | |
| Yes | 10 (43.5) |
| Yes, partly | 5 (21.7) |
| No, but I am completing this survey on behalf of someone else | 7 (30.4) |
| Do not know/unsure | 1 (4.4) |
| Missing data | 0 |
| Table E1 describes the sociodemographic data of the survey respondents. | |

| **eTable 3. Characteristics of reporting hospitals and units or wards**.  Data presented are n and (%) for 91 units. | |
| --- | --- |
| **Hospital type** | |
| Community hospital | 48 (52.8) |
| University-related/aﬀiliated hospital | 30 (33.0) |
| University hospital | 12 (13.2) |
| Private hospital | 1 (1.1) |
| Nursing home | 0 (0) |
| Rehabiliation center | 0 (0) |
| Other | 0 (0) |
| **Numbers of beds** | |
| <250 | 29 (31.9) |
| 250-499 | 17 (18.7) |
| 500-749 | 31 (34.1) |
| 750-999 | 13 (14.3) |
| 1000-1499 | 1 (1.1) |
| ≥1500 | 0 (0) |
| **Discipline** | |
| Mixed/general | 33 (36.3) |
| Medical/non-surgical | 32 (35.2) |
| Surgical | 19 (20.9) |
| Other | 6 (6.6) |
| Rehabilitation | 1 (1.1) |
| Long care | 0 (0) |
| Respiratory/weaning | 0 (0) |
| Palliative | 0 (0) |
| **Ward/unit type** | |
| High acuity, IMC, ICU (categorized as ICU) | 58 (63.7) |
| General ward | 31 (34.1) |
| Emergency Department | 1 (1.1) |
| Other | 1 (1.1) |
| Rehabilitation facility | 0 (0) |
| Nursing home | 0 (0) |
| **Years of clinical experience on the reporting unit** | |
| <5 | 32 (35.2) |
| <10 | 16 (17.6) |
| <15 | 32 (35.2) |
| <20 | 3 (3.3) |
| ≥20 | 8 (8.8) |
| eTable2 describes the survey responses detailing reporting hospitals and the specific unit or ward. | |

| **eTable 4. Descriptors of primary outcome, clinically documented delirium prevalence**  Data presented are n and (%) for 91 units. | |
| --- | --- |
| **How often is delirium assessed** |  |
| Twice per 24 hr | 65 (72.2) |
| > thrice per 24 hr | 13 (14.4) |
| Thrice per 24 hr | 5 (5.6) |
| Only if sudden changes of consciousness | 3 (3.3) |
| Once per 24 hr | 2 (2.2) |
| Other | 2 (2.2) |
| Only at admission | 0 (0) |
| **Person primarily responsible for assessment** | |
| Nurse | 89 (97.8) |
| Psychologist/psychiatrist | 2 (2.2) |
| Physician | 0 (0) |
| Mixed professions | 0 (0) |
| None | 0 (0) |
| Geriatrician | 0 (0) |
| Specific delirium team | 0 (0) |
| Other | 0 (0) |
| Table E3 summarizes the frequency of delirium assessment and the profession primarily responsible for conducting these assessments. | |

| **eTable 5. Types of validated and non-validated delirium assessments used.**  Data presented are n and (%) for 91 units. | |
| --- | --- |
| **Validated assessments** | **All** |
| CAM-ICU | 48 (52.8) |
| CAM | 17 (18.7) |
| Nu-DESC | 9 (9.9) |
| bCAM | 5 (5.5) |
| DTS | 3 (3.3) |
| 4AT | 2 (2.2) |
| Psychiatric consult | 2 (2.2) |
| CAM-ICU-7 | 1 (1.0) |
| ICDSC | 0 (0) |
| CAP-D | 0 (0) |
| DSM-V criteria | 0 (0) |
| DSM-IV criteria | 0 (0) |
| DSM-VI criteria | 0 (0) |
| 3D-CAM | 0 (0) |
| SQID | 0 (0) |
| SOS-PD | 0 (0) |
| pCAM-ICU | 0 (0) |
| PAED Scale | 0 (0) |
| UB-CAM | 0 (0) |
| **Non-validated assessments** | |
| Personal judgement | 4 (4.4) |
| None | 0 (0) |
| Other (valid and non-valid combined) | 0 (0) |
| The survey responses received to the question “What type of delirium assessments do you use on this ward or unit? (In case of multiple assessments, tick all that apply) are displayed in Table E4.  Abbreviations*:* 3DCAM: 3-Minute Diagnostic Confusion Assessment Method; 4AT: 4 ‘A’s Test (Arousal, Attention, Abbreviate Mental Test – 4, Acute change); bCAM: Brief Confusion Assessment Method; CAM: Confusion Assessment Method; CAM-ICU: Confusion Assessment Method for the Intensive Care Unit; CAM-ICU-7: Confusion Assessment Method Intensive Care Unit Severity Score; CAP-D: Cornell Assessment of Pediatric Delirium; DSM-IV criteria: Diagnostic and Statistical Manual of Mental Disorders, Fourth Edition; DSM-V criteria: Diagnostic and Statistical Manual of Mental Disorders, Fifth Edition; DSM-VI criteria: Diagnostic and Statistical Manual of Mental Disorders, Sixth Edition; DTS: Delirium Triage Screen; ICDSC: Intensive Care Delirium Screening Checklist; Nu-DESC: Nursing Delirium Screening Scale; pCAM-ICU: Pediatric Confusion Assessment Method for the Intensive Care Unit; psCAM-ICU: Preschool Confusion Assessment Method for the Intensive Care Unit; SOS-PD: Sophia Observational withdraw Symptoms-Paediatric Delirium Scale; SQiD: Single Question in Delirium; UB-CAM: Ultra Brief Confusion Assessment Method | |

| **eTable 6. Potential future projects generated from WDAD 2023 survey & U.S. study team** | |
| --- | --- |
| Project Idea | Description |
| Identification & development of key metrics | Goal: To track progress of delirium-focused implementation or quality improvement projects.  Definition: These data-based key metrics must convey the accomplishment of meaningful progress towards evidence-based delirium care, and proactively identify barriers or gaps in care that need to be address. |
| Development of quality improvement toolkits | Goal: To support adoption of evidence-based delirium are at the unit level |
| Partnership with existing delirium societies | Goal: To train clinical staff on the conduct of quality improvement projects at the unit level |
| Address pandemic-related burnout | Goal: To improve staff retention and work environment, interventions to address pandemic-related emotional distress, post-traumatic stress syndrome, and moral distress such as peer-to-peer support groups should be instituted at the unit-level.^1,2^ |
| Interdisciplinary/interprofessional approach | Goal: To increase the amount of interdisciplinary and interprofessional team members to improve the approach to delirium assessment and management in clinical care |
| Integrate WDAD survey as a measure of change | Goal: To provide a toolkit of templated resources for an institution to deploy. The toolkit would include guidance on how to share results and next steps with hospital administration and unit/hospital members to gain support, frameworks for practice change, dashboards to monitor practice change and human behavior, and guidance on how to develop a minimal standard operating procedure (MSOP). The toolkit could be tailored, or localized, to each unique unit. This process would start with administration of the WDAD survey to assess status of delirium care practices within the institution. Guidance on how to use the survey results as a comparison to current practice would be included. |
| eTable 6 describes the ideas for future projects generated from the U.S. 2023 WDAD study team. These ideas were informed by previous and current experience implementing delirium-related practice changes. | |

References

1. McAndrew NS, Rosa WE, Moore KM, et al. Sprinting in a Marathon: Nursing Staff and Nurse Leaders Make Meaning of Practicing in COVID-19 Devoted Units Pre-Vaccine. *SAGE Open Nurs*. Jan-Dec 2023;9:23779608231165688. doi:10.1177/23779608231165688

2. Guttormson JL, Calkins K, McAndrew N, Fitzgerald J, Losurdo H, Loonsfoot D. Critical Care Nurses' Experiences During the COVID-19 Pandemic: A US National Survey. *Am J Crit Care*. Mar 1 2022;31(2):96-103. doi:10.4037/ajcc2022312
